# The effects of treatment on lipoprotein subfractions evaluated by polyacrylamide gel electrophoresis in patients with autoimmune hypothyroidism and hyperthyroidism

**DOI:** 10.1186/1476-511X-13-158

**Published:** 2014-10-10

**Authors:** Zuzana Minarikova, Ludovit Gaspar, Peter Kruzliak, Zuzana Celecová, Stanislav Oravec

**Affiliations:** 2nd Department of Internal Medicine, Faculty of Medicine, University Hospital and Comenius University, Bratislava, Slovak Republic; Department of Cardiovascular Diseases, International Clinical Research Center, St. Anne’s University Hospital and Masaryk University, Pekarska 53, Brno, 656 91 Czech Republic; Department of Internal Medicine, Hospital of L. N. Jégé, M.D, Dolný Kubín, Slovakia; Krankenanstalten Labor Dr. Dostal, Vienna, Austria

**Keywords:** Atherogenic dyslipidemia, Hypothyroidism, Small dense LDL, apoB, Atherogenic index of plasma

## Abstract

**Background:**

Atherogenic dyslipoproteinemia is one of the most important risk factor for atherosclerotic changes development. Hypothyroidism is one of the most common causes of secondary dyslipidemias which results from reduced LDL clearance and therefore raised levels of LDL and apoB. Association between small dense LDL (sdLDL) presentation and thyroid status has been examinated using polyacrylamide gel electrophoresis for lipoprotein subfractions evaluation.

**Methods:**

40 patients with diagnosed autoimmune hypothyroidism and 30 patients with autoimmune hyperthyroidism were treated with thyroxine replacement or thyreo-suppressive treatment. In both groups lipid profiles, LDL subractions, apolipoproteins (apoA1, apoB), apoA1/apoB ratio and atherogenic index of plazma (AIP) were examined before treatment and in state of euthyreosis.

**Results:**

Thyroxine replacement therapy significantly reduced levels of total cholesterol (TC), LDL, triglycerides (TG) and also decreased levels of sdLDL (8,55±11,671 vs 0,83±1,693mg/dl; p<0,001), apoB and AIP. For estimation of atherogenic lipoprotein profile existence an AIP evaluation seems to be better than apoB measurement because of the more evident relationship with sdLDL (r=0,538; p<0,01). Thyreo-suppressive therapy significantly increased levels of TC, LDL, TG and apoB. The sdLDL was not found in hyperthyroid patients.

**Conclusions:**

Atherogenic lipoprotein profile was present in 52.5% of hypothyroid subjects, which is higher prevalence than in normal, age-related population. Substitution treatment leads to an improvement of the lipid levels, TG, apoB, AIP and LDL subclasses. It significantly changed the presentation of sdLDL – we noticed shift to large, less atherogenic LDL particles. Significantly positive correlation between sdLDL and TAG; sdLDL and VLDL alerts to hypertriglyceridemia as a major cardiovascular risk factor.

## Background

The relationship between small dense LDL (sdLDL) and coronary heart disease (CHD) has been a debated issue for a long time [1]. Many cross-sectional studies have confirmed the finding of Fisher et al. [2]. They first pointed out that among patients with CHD (with premature atherosclerosis) “diverse” LDL are more present in comparison to the control group [2, 3]. Results of cross-sectional and prospective epidemiological studies point to the fact that the predominance of sdLDL in plasma is associated with the development of CHD [1, 4, 5]. This finding has been accepted as a warning cardiovascular risk factor by the National Cholesterol Education Program Adult Treatment Panel III (NCEP-ATP III) [6].

Elevated levels of the sdLDL may increase the risk of atherosclerosis in various levels of LDL. Thanks to specific properties of sdLDL predominance of sdLDL means increased risk of atherogenesis in comparison to increased levels of LDL1 and LDL2 [7, 8]. SdLDLs have easier access to the subendothelial spaces in the arterial wall and exhibit enhanced binding to intimal proteoglycans [7, 9, 10]. This increased risk of atherogenesis may be partially due to increased susceptibility to oxidation and glycation or to decreased clearance becouse of reduced affinity for the LDL receptor [7, 11]. SdLDLs also exhibit increased uptake by macrophages, therefore facilitating the formation of foam cells [12]. Furthermore, sdLDL has slowed metabolism compared to that of medium-sized LDL [13] and is associated with an elevated fibrinogen level [14]. Finally, an inverse relationship between LDL particle size and level of plasminogen activator inhibitor-1, a factor associated with impaired fibrinolysis and atherosclerotic disease [15]. This makes from sdLDL strong atherogenic lipoprotein [5, 7, 16, 17]. For these reasons the size determination of the LDL particles, is an important step in cardiovascular risk assessment, along with use of other certificated parameters.

The recent knowledge of LDL subclasses have an impact on therapy not only primary but also secondary dyslipidemias. Thyroid diseases, like hypothyroidism, represent one of the most common types of secondary dyslipidemias. The examination of lipoproteins as well as the possibility of LDL subclasses analysis can provide a different perspective and better evaluation of cardiovascular risk in these patients. There are not many publications concerning this topic in the literature and moreover present contradictory results. Other reports are more focused on standard indicators of atherogenesis such as total cholesterol (TC), LDL cholesterol, apolipoprotein B (apoB), triglycerides (TG), as on the evaluation of LDL subclasses [18–24].

Ultimately the results suggest that decreased thyroid function affects the size of LDL and the center of clinical interest is again in the metabolism of triglycerides, hypertriglyceridemia, and their potential to shape the atherogenic lipoprotein subpopulations [19, 22]. Because of suggestions that thyroid hormones modulate serum lipoprotein levels, we aimed to assess the relationship between LDL particle size (included in lipoprotein spectrum) and thyroid function changes in selected groups of patients. We decided to evaluate a relationship between atherogenic dyslipidemia, hypothyroidism and hyperthyroidism and the effect of treatment on the lipoprotein spectrum in these patients.

## Methods

There were two groups of participants attending the Endokrinology outpatient clinic of 2nd Department of Internal Medicine in University Hospital in Bratislava, Slovakia and also the Private endocrinology outpatient clinic of M. Podobová, MD. in ENDOTOPMED s.r.o. Bratislava, Slovakia. In the first group forty newly diagnosed patients with autoimmune hypothyroidism (36 women, 4 men) were included. The second group comprised thirty newly diagnosed patients with autoimune hyperthyroidism (25 women, 5 men). Patients were classified as hypothyroid if serum TSH level was > 5 mIU/l or hyperthyroid if serum TSH level was 0,5 < mIU/l, taking into consideration fT4 levels as well. The reference range in our laboratory is: TSH (0.55-4.78 mIU/l); fT4 (11.5 – 22.7 pmol/l). Exclusion criteria were: diabetes mellitus, other diseases of the endocrine system, familiar hypercholesterolemia, liver or renal disease, pregnant women, patients with stroke, heart attack, cancer, pancreatitis, alcohol abuse and use of known lipid-lowering drugs. All patients were recruited into the study voluntarily after signing informed consent. The research proposal was approved by the Ethical Committee of the University Hospital in Bratislava and Faculty of Medicine of Comenius University in Bratislava, Slovakia.

### Blood samples

Blood samples were collected, from all participants subjects after 12 hours overnight fasting and stored in plain tubes. Serum was isolated after clot retraction and centrifugation (3500 rev/min, 15 min) and subsequently divided into 3 aliquot parts and stored at -70°C until analysis was performed within 1 month of sample collection. Other tubes for biochemical analysis were immediately sent to the laboratory for examination.

### Assays

Basic laboratory biochemical examination: total cholesterol (TC): CHOD-PAP enzymatic method (cholesterol oxidase - 4-aminoantipyrine); triglycerides (TG), - an enzymatic GPO-PAP method (glycerol-3-phosphate oxidase - 4-aminoantipyrine); LDL cholesterol - the direct selective enzymatic determination; HDL cholesterol - the direct immuno-inhibitory enzymatic determination; (Erba-Lachema, Brno, ČR).

For the thyroid function tests: TSH, fT4 - chemiluminescence immunoassay (LIA, Siemens); for autoantibodies against thyroid tissue (aTg, aTPO eventually aTSHR) and thyroglobulin (Tg) - electrochemiluminescence immunoassay (ECLIA, Roche).

Apolipoprotein A1 and apolipoprotein B serum were examinated by imunoturbidimetric method (Roche, SRN). ApoA1/apoB ratio and AIP (log (TAG/HDL cholesterol) were evaluated manually [25, 26].

### LDL subclass analysis

To identify atherogenic lipoproteins several methods (gradient gel electrophoresis, proton magnetic resonance spectroscopy, ultracentrifugation) have been developed. They are, however to laborious, expensive and not appropriate for routine clinical use [27–30]. An alternative and less demanding approach to LDL subclasses study is a modified tube gel electrophoresis technique which has been commercialized on the Lipoprint system (Quantimetrix Corporation, USA) [31]. It is relatively simpler to operate, provides particle size, allows quantitation of sdLDL fractions and correlates with the other methods [31–33].

This method enables the analysis of 12 lipoprotein subfractions: VLDL; IDL1, IDL2, IDL3 (MID C, B, A); LDL1, LDL2 (large buoyant LDL), LDL 3–7; and HDL. According to LDL electrophoretic profile, 2 phenotypes can be defined: phenotype A with normal total cholesterol mass of the sdLDL subfractions and phenotype non-A (called phenotype B) where total cholesterol mass of the sdLDL subfractions is intermediate – low [11, 31–35]. Atherogenic lipoprotein profiles are characterized by a predominance of atherogenic lipoproteins, namely very low density (VLDL), intermediate density IDL1 and IDL2 and particularly by the presence of small dense lipoproteins with low density (LDL). These profiles identify highly atherogenic LDL subfractions - the LDL 3–7 fractions. These subfractions are smaller, with a diameter < 26.5 nm (265 Angström) and they float within a density range of 1.048 – 1.065 g/ml, a higher density than LDL1 and LDL2 [7, 11, 17]. The cutoff for elevated cholesterol mass of the sdLDL subfraction with this system is 0.155 mmol/l [35]. The size cutoff is above or equal to 26,8 nm for phenotype A (normal LDL size) and less than the mentioned value for non-A (phenotype B) [19, 32]. Interpretation and determination of non-atherogenic Type A eventually atherogenic Type B lipoprotein spectrum is performed by software for Lipoprint LDL system (Lipoprint LDL system, Quantimetrix Corporation, USA) [31].

The process involves applying 25 μl of serum sample to the „redy-to-use” polyakrylamide gel tube with 200 μl of a loading high-resolution 3% gel solution containing a lipophilic dye (Sudan Black B). The dye binds to the cholesterol in the lipoprotein particles permitting the visualization and measurement of lipoprotein fractions after electrophoretic separation. The photo-polymerization of sample loading gel mixture happens at room temperature for 30 min and is followed by electrophoresis at a constant current of 3 mA/elfo-tube for one hour. In a series of 12 samples, one represents a control sample with a defined value of the concentration of lipoproteins and their subpopulations and is part of LipoprintLDL set. When the process of electrophoresis is finished, tubes are left in the dark for 10 minutes. The next step is densitometric evaluation of the tubes (610 nm) [31].

Serum lipoprotein sub-populations (VLDL, MID A-B (IDL 3–1), LDL 1–7, HDL) are divided on the basis of particles size. VLDL migrates as the slowest (Rf = 0) and can be identified on a strip at the beginning of the gel. MID and LDL migrate between Rf = 0 and Rf = 1. HDL particles are the smallest, migrate farthest and form a band at the forefront of the gel, Rf = 1 [31]. Gel images are analyzed by software for Lipoprint LDL system (Lipoprint LDL system, Quantimetrix Corporation, USA). The bands are divided into discrete segments and the relative area under the curve is calculated for each lipoprotein band. Also cholesterol concentration for each lipoprotein fraction is calculated by this program using total cholesterol value obtained for each sample by the method indicated previously [19, 31–33].

All tested parameters were measured in both groups, in patients with newly diagnosed autoimmune hypothyroidism and hyperthyroidism, before treatment and also in euthyreosis, after thyreo-substitution or after thyreo-suppressive therapy.

### Types of dyslipoproteinemias according to lipoprotein phenotype

**Figure 1:**
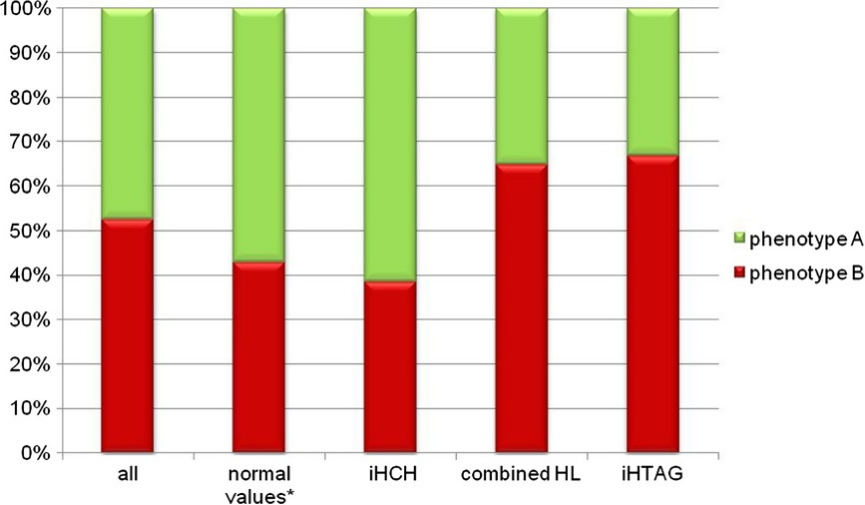
**Phenotype A and B in hypothyroid patients.** * - normal values of TC, LDL, TG; iHCH – isolated hypercholesterolemia; combined HL – combined hyperlipidemia; iHTAG – isolated hypertriglyceridemia.

Among patients with hypothyroidism 32.5% had isolated hypercholesterolemia, 42.5% had combined hyperlipidemia and 7.5%, had isolated hypertriglyceridemia. The rest (17.5%) had normal lipids levels. Phenotype B occured in 21 participants (52.5%), which was more often in the case of combined hyperlipidemia and hypertriglyceridemia than in isolated hypercholesterolemia or in subjects with normal lipid levels (Figure 1). Hyperthyroid patients did not have hyperlipoproteinemia. Atherogenic lipoprotein profile type B was not present in these patients. Non-atherogenic lipoprotein profile was detected in all patients in this group. All the participants had normal levels of lipids.

### Data analysis

Statistical analysis was performed using SPSS for Windows 20.0 (Student’s *t*-test; unpaired *T*-test; Kolmogorov – Smirnov test; Pearson corelation; Spearman corelation; Analysis of variance (ANOVA); Kruskal – Wallis test; Wilcoxon signed-rank test). Descriptive statistics were used to present the data. Non-parametric statistical methods were used as the variables were not normally distributed. P-value < 0.05 was considered as significant.

## Results

Anthropometric and demographic features of the participants are shown in Table 1. Patients with hypothyroidism were treated with levothyroxine. The average dose of levothyroxine was 81.25 g/day (50-150 μg). The appropriate dose was necessary to be individually adjusted during therapy. The substitution treatment lasted on average for 7.13 months (SD = ± 2.32 months, range 3–12 months). Patients with hyperthyroidism were *suppressed* by the *treatment with* either thiamazole (doses up to 40 mg/day) or propylthiouracyl (doses up to 100 mg/day). The starting dose was higher than the maintenance dose and it was also necessary to adjust the unit dose individually to patient. In some cases it was required to go for the combination therapy or change the treatment. Most patients were treated with thiamazole, especially towards to the end of the study. The therapy lasted on average of 9.35 months (SD = ± 5.84 months, range: 2–21 months).

**Table 1:** **Characteristics of study participants**

Group	Hypothyroidism		Hyperthyroidism	
	*Min-Max*	*Average ± SD*	*Min-Max*	*Average ± SD*
**Age, years**	32-82	61.7 ± 12.03	28-58	43.9 ± 9.26
**BMI, kg/m** ^**2**^	19.61-37.78	27.19 ± 4.1	19.66- 30.86	24.68 ± 3.51
**Smokers, n(%)**	4 (10%)		5 (16.67%)	
**Sex, F/M**	36/4		25/5	

BMI – Body Mass Index.

### Correlations of plasma atherogenic markers

To find out the correlation relationship between variables, we used Pearson's correlation coefficient. Subsequently we used the Spearman correlation coefficient to determine the statistical significance between non-parametric data. The correlations between subpopulations of LDL, VLDL and HDL compared with AIP, apoB and apoA1/apoB ratio in a group of patients with hypothyroidism are shown in Table 2. There was significantly positive correlation between apoB and LDL3-7, AIP and LDL3-7 and both apoB and AIP with VLDL in hypothyroid patients. On the other hand we determined the positive correlation between apoB and LDL2. ApoB levels were significantly higher also in the presence of IDL lipoproteins (IDL1: r = 0.386; p < 0.05: IDL2 r = 0.436; p < 0.01). AIP positively correlated with apoB too (r = 0.422, P <0.01).

**Table 2:** **Correlations between subpopulations of LDL, VLDL and HDL compared with AIP, apoB and apoA1/apoB ratio in a group of patients with hypothyroidism**

	AIP	apoB	apoA1/apoB
	r/ρ		
**Total LDL**	0.169^(NS)^	0.706**	-0.431**
**LDL 1**	-0.186^(NS)^	0.264^(NS)^	-0.049^(NS)^
**LDL 2**	0.211^(NS)^	0.646**	-0.482**
**LDL 3-7**	0.538**	0.570**	-0.635**
**VLDL**	0.545**	0.571**	-0.329*
**HDL**	-0.491**	0.111^(NS)^	0.543**

r/ρ: r - Pearson’s ev. ρ - Spearman’s correlation coefficient.

(** = P <0.01, * = p <0.05, ^NS^ = not significant).

The relationships between selected groups of lipoproteins and TG in a group of patients with hypothyroidism are shown in Table 3. The presence of LDL3-7 is not dependent on the TC and total LDL levels in patients with hypothyroidism. There was a significantly positive correlation between LDL3-7 and serum TG, LDL2, and also with VLDL. IDL1 significantly positively correlated with TG (r = 0.356, P < 0.01). Conversely HDL positively correlated with serum non-atherogenic lipoproteins such as LDL1 and IDL3 (r = 0.461, P < 0.01). We also recorded a positive correlation between TG levels and BMI in our study (r = 0.329, p < 0.05).

**Table 3:** **Relationships between selected groups of lipoproteins in a group of patients with hypothyroidism**

	VLDL	LDL1	LDL2	LDL3-7
	r/ρ			
**TC**	0.572**	0.640**	0.608**	0.311^(NS)^
**LDL**	0.362*	0.698**	0.701**	0.294^(NS)^
**HDL**	0.008 ^(NS)^	0.441**	-0.091 ^(NS)^	-0.367*
**TAG**	0.561^**^	0.111 ^(NS)^	0.270 ^(NS)^	0.464**
**LDL 3-7**	0.342^*^	-0.186^(NS)^	0.627**	-

r/ρ: r - Pearson’s ev. ρ - Spearman’s correlation coefficient.

(** = P <0.01, * = p <0.05, ^NS^ = not significant).

Except for LDL3-7, thyroid function parameters were also identified as nonparametric data. We didn’t found any correlation relationship between TSH and LDL3-7 (ρ = -0.069, NS) or between fT4 and LDL3-7 (ρ = -0.153, NS) using Spearman correlation coeficient.

### The effects of treatment on lipoprotein subfractions

The comparison of the data at the time of diagnosis and after induction of euthyreosis was made by Student’s paired *t*-test. For non-parametric data we then used Wilcoxon signed-rank test. Input and after treatment values for thyroid function parameters, lipid, apolipoprotein levels, apoA1/apoB, AIP and lipoprotein subclasses for hypothyroid patients are shown in Table 4.

**Table 4:** **Input and after treatment values for thyroid function parameters, lipid, apolipoprotein levels, apoA1/apoB ratio, AIP and lipoprotein subclasses for hypothyroid patients**

	Hypothyroidism						
	Before treatment			In euthyreosis			
	*Average*	*±SD*	*Min-Max*	*Average*	*±SD*	*Min-Max*	*Signif.*
**TC (mmol/l)**	6.10	±1.56	2.6-9.2	5.45	±1.03	3.26-7.27	**
**TG (mmol/l)**	2.05	±1.62	0.7-10.1	1.29	±0.55	0.48-2.5	***
**LDL (mmol/l)**	3.86	±1.25	1.6-6.4	3.38	±0.85	1.89-5.33	**
**HDL (mmol/l)**	1.21	±0.31	0.6-2	1.26	±0.31	0.67-1.99	NS
**apoA1(g/l)**	1.89	±0.65	0.87-3.47	1.67	±0.40	0.77-2.6	*
**apoB (g/l)**	1.02	±0.29	0.41-1.64	0.92	±0.21	0.56-1.5	**
**apoA1/apoB**	1.95	±0.71	0.87-3.44	1.86	±0.5	1.16-2.88	NS
**AIP**	0.17	±0.28	-0.31-0.95	-0.02	±0.26	-0.38-0.47	***
**LDL1 (mg/dl)**	47.35	±18.48	10-113	53.78	±15.33	32-87	*
**LDL2 (mg/dl)**	33.85	±19.53	0-76	20.48	±11.25	5-53	***
**IDL1 (mg/dl)**	24.18	±10.76	8-50	20.93	±6.84	10-36	*
**IDL2 (mg/dl)**	15.78	±6.88	4-30	13.35	±3.87	8-21	*
**IDL3 (mg/dl)**	19.38	±9.54	4-43	21.23	±5.21	13-32	NS
**VLDL (mg/dl)**	40.03	±16.84	8-80	31.20	±9.58	10-61	***
**Not-normally distributed variables:**							
	*Median*	*Q3-Q1*	*Min-Max*	*Median*	*Q3-Q1*	*Min-Max*	*Signif.*
**TSH (mIU/l)**	19.6	27.04	10.57-150	3.21	3,79	0.5-4.8	***
**fT4 (pmol/l)**	7.24	3.17	1.56-9.0	15	3,41	12-21.8	***
**LDL3-7 (mg/dl)**	6.5	10	0-54	0	1	0-8	***

apo – apolipoprotein, AIP – atherogenic index of plasma, *Q3-Q1 –* interquartile range.

* significantly different via the Student’s *t*-test or Wilcoxon signed-rank test for non-parametric data: * p < 0.05; ** p < 0.01; *** p < 0.001

**Figure 2:**
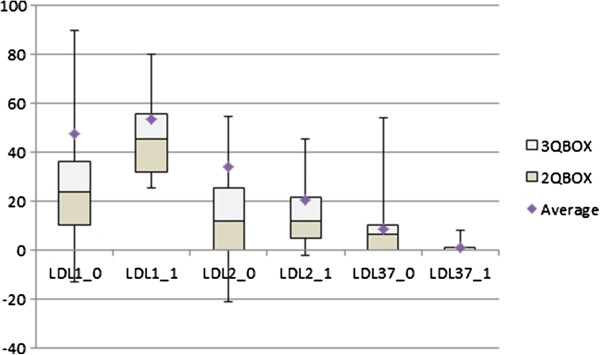
**The Effect of treatment on LDL subfractions in hypothyroid patients.** (0) before treatment (1) in euthyreosis.

Thyroxine substitution significantly reduced serum lipids, except HDL. All patients had a phenotype A in euthyreosis. Hormone replacement therapy also significantly decreased serum levels of apolipoprotein B. There was a significant reduction in AIP and no significant change in values of apoA1/apoB ratio. Levels of LDL3-7 was significantly reduced in euthyreosis as well as were the other lipoprotein subpopulations (Table 4, Figure 2). On the other hand, there was a significant increase in LDL1 levels. An important finding was the after-treatment identification of the hyper-beta-lipoproteinemia with LDL 1,2 non-atherogenic lipoprotein profile, phenotype A (30% of subjects). This type of lipoprotein spectrum was found also prior to thyroxine substitution (15%). Even if the patient with hypothyroidism had no atherogenic lipoprotein spectrum, also in this case the medical thyroid therapy decreased individual levels of lipoproteins.

**Figure 3:**
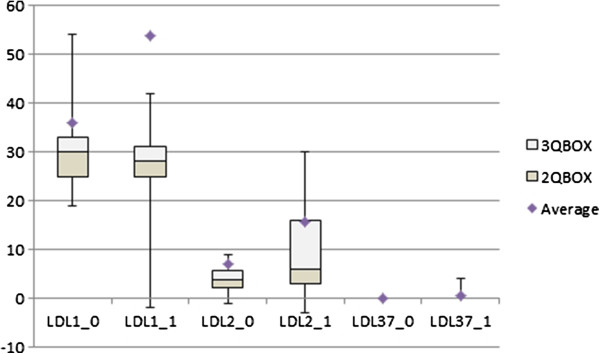
**The Effect of treatment on LDL subfractions in hyperthyroid patients**. (0) before treatment (1) in euthyreosis.

Input and after treatment values for thyroid function parameters, lipids, apolipoprotein levels, apoA1/apoB ratio, AIP and lipoprotein subclasses for hyperthyroid patients are shown in Table 5, Figure 3. Suppressive therapy for thyroid significantly elevated levels of serum lipids and individual lipoprotein subclasses. This treatment also significantly increased levels of apolipoproteins. Their ratio (apoA1/apoB) was significantly reduced. There was not a significant increase in AIP values. In euthyroid state TG levels, LDL and HDL cholesterol were also in the reference range. There was only a light increase in TC levels. The average value of TC was slightly higher than the upper limit of the reference range (5.571 ± 1.079 mmol/l). Also in euthyreosis all patients had phenotype A and there were 16.67% of participants with hyper-beta-lipoproteinemia with LDL 1,2 non-atherogenic lipoprotein profile, phenotype A.

**Table 5:** **Input and after treatment values for thyroid function parameters, lipid, apolipoprotein levels, apoA1/apoB ratio, AIP and lipoprotein subclasses in hyperthyroid patients**

	Hyperthyroidism						
	Before treatment			In euthyreosis			
	*Average*	*±SD*	*Min-Max*	*Average*	*±SD*	*Min-Max*	*Signif.*
**TC (mmol/l)**	3.65	±0.62	2.9-5.0	5.57	±1.08	2.77-6.9	***
**TG (mmol/l)**	1.0	±0.29	0.6-1.6	1.22	±0.37	0.58-1.81	**
**LDL (mmol/l)**	2.05	±0.42	1.47-3.21	3.26	±0.77	1.34-4.29	***
**HDL (mmol/l)**	1.03	±0.22	0.67-1.45	1.38	±0.26	0.93-2.17	***
**apoA1 (g/l)**	1.56	±0.29	1.10-2.09	1.92	±0.25	1.52-2.6	***
**apoB (g/l)**	0.61	±0.12	0.42-0.99	0.92	±0.17	0.42-1.15	***
**apoA1/apoB**	2.59	±0.55	1.90-3.76	2.16	±0.49	1.58-3.79	***
**AIP**	-0.02	±0.20	-0.33-0.32	-0.07	±0.17	-0.50-0.18	NS
**LDL1 (mg/dl)**	35.97	±8.29	25-60	53.80	±10.75	25-69	***
**LDL2 (mg/dl)**	7.00	±2.42	2-12	15.67	±10.17	3-36	***
**IDL1 (mg/dl)**	11.93	±2.53	6-17	17.33	±5.38	8-26	***
**IDL2 (mg/dl)**	8.90	±2.58	5-14	12.17	±3.87	4-18	***
**IDL3 (mg/dl)**	15.47	±4.97	7-27	26.63	±8.27	5-42	***
**VLDL (mg/dl)**	22.27	±9.32	12-43	35.83	±9.51	20-55	***
**Not-normally distributed variables:**							
	*Median*	*Q3-Q1*	*Min-Max*	*Median*	*Q3-Q1*	*Min-Max*	*Signif.*
**TSH (mIU/l)**	0.001	0.0066	0.0025-0.054	2.1	2,22	0.5-4.9	***
**fT4 (pmol/l)**	55.9	42,28	25.07-138	16.4	4,38	12.1-21.84	***
**LDL3-7 (mg/dl)**	0	0	0	0	0	4	*

apo – apolipoprotein, AIP – atherogenic index of plasma, *Q3-Q1 –* interquartile range.

* significantly different via the Student’s *t*-test or Wilcoxon signed-rank test for non-parametric data: * p < 0.05; ** p < 0.01; *** p < 0.001.

## Discussion

Cardiovascular diseases (CVD) remain the major cause of morbidity and mortality in the world. Atherogenic dyslipoproteinemia which is involved in the formation of either macro- or microvascular changes is now considered as the most important risk factor for atherosclerotic changes development [36–41]. An association betwen hypothyroidism and coronary hearth disease (CHD) has been shown in many epidemiological studies [42–46]. The induction of atherogenic dislipidaemia seems to be a major component of the association between hypothyroidism and CHD risk [19].

It is well known, that LDL represent a heterogeneous group of particles which differ in size (22–27.2 nm), density, cholesterol, and apoB conformation [1, 3, 4, 7, 36]. Nondenaturing gradient gel electrophoresis can identify as many as seven distinct subspecies of LDL based on density, that are grouped in four major subclasses LDL-I-IV, from the largest, most buoyant to the smallest, most dense particles [7]. In an alternative classification based on particle diameter Austin *et al.* [47] suggested two major patterns of LDL profile, pattern A (particle diameter 25.5 nm or greater) and pattern B (particle diameter less than 25.5 nm). It seems likely that LDL atherogenicity increases continuously with decreasing particle size and therefore that in this population small LDLs will tent to be more atherogenic than large LDLs [19]. Two phenotypes have been identified (i) phenotype A comprising large, less dense LDL, present at low TG levels; and (ii) phenotype B with abundant sdLDL coupled with elevated TG and decreased HDL [7, 19].

In our study we would like to point out to the identification of the hyper-beta-lipoproteinemia, with LDL1, 2 non-atherogenic lipoprotein profile A. This type of lipoprotein spectrum was found, either in a group of patients with hypothyroidism prior to treatment (15%) or in some patients with euthyreosis after the treatment in both groups (46.67%). LDL1 and LDL2 do not fulfil the criteria of atherogenicity for lipoprotein entities which are usually ascribed to LDL lipoproteins. Oravec et al. reported, that LDL1 and LDL2 subfractions in hypercholesterolemic indidviduals which created a non-atherogenic hyper-betalipoproteinemia LDL1,2 without the presence of atherogenic sdLDL were associated with a high concentration of cardiovascular protective HDL subfractions [48]. Although LDL2 particles are classified as a large, buoyant LDL particles, their non-atherogenic effect is possibly unclear. In our study we found positive correlation between LDL2 levels and LDL3-7 and also apoB (Table 2, 3). On the other side Castelli published evidence, that more than 75% of the patients with an acute coronary syndrome or a myocardial infarction had normal plasma values of LDL cholesterol or HDL cholesterol [49–51]. In our study we also identified normolipemic patients with atherogenic lipoprotein profile. In this cases, the methods enabling precise identification of atherogenic lipoprotein subpopulations with their clear quantitation are reasonable and warranted.

We have also focused on the relationship between plasma indicators of atherogenesis (Table 2). Results shows significant positive correlation between AIP, apoB and sdLDL (LDL3-7). ApoB were significantly higher not only in the presence of elevated LDL3-7, but even in the presence of LDL2 or IDL lipoproteins. ApoB100 is present in all lipoprotein subclasses of LDL, VLDL, IDL, and also Lp (a). ApoB levels provide an indirect information of particle density and size in those patients, where we can assume an overgrowth of sdLDL [16]. Also other studies indicate that elevated apoB are not necessarily directly involved in sdLDL pathogenesis in hypothyroidism [19]. However, AIP (log TG/HDL-C) provides an information about the lipoprotein profile in only one value. AIP allows the prediction of the size of individual particles. Its value significantly correlated with the size of LDL, HDL and VLDL particles. It’s an indicator of atherogenic lipoprotein phenotype (high risk >0.21; low risk < 0.11; intermediate risk 0.11-0.21) [25, 26]. There was positive correlation between AIP adn LDL3-7 and also AIP and VLDL in our study. For estimation of atherogenic lipoprotein profile existence, an evaluation of the AIP seems to be better than apoB measurement. Unfortunately this parameter has its limitations in patients with familial hypercholesterolemia, hypertriglyceridemia or chylomicronemia [52].

Relationship between TG levels as a resonable predictor of LDL size phenotype category was documented in several studies [19, 47]. In this context, also in our study phenotype B occurs more often in the case of combined hyperlipidemia and hypertriglyceridemia than in isolated hypercholesterolemia or in subjects with normal lipid levels. TG represent an important biomarker of CVD risk becouse of their association with atherogenic remnant particles and apoCIII, a proinflamatory, proatherogenic protein found on all classes of the plasma lipoproteins. Hypertriglyceridemic states are associated with increased VLDL production and delayed VLDL clearance from circulation [53–55]. Small dense LDL tend to be the rule with triglycerides >1.5 mmol/l [11, 19, 56] and sdLDL particles are usually found in association with high TG and low HDL. The mechanism of this association could be explained by the increased of VLDL formation in elevated TG levels, that result in TG enriched and cholesterol ester depleted LDL particles (with effect of CEPT – cholesterol ester transfer protein). Hepatic lipase than cleaves out the TG leaving cholesterol ester depleted LDL [17] which are physically smaller and becouse of resultant relative increase in protein also denser [19]. This idea is also supported by the results in our study (Table 3). There was a significantly positive correlation between LDL3-7 and serum TG levels; and also between LDL3-7 and VLDL levels. These findings remind us that hypertriglyceridemia is one of the major cardiovascular risk factors, because of the VLDL1 from which highly atherogenic LDL subfractions are formed, and may also be used to follow the treatment. Association between hypertriglyceridemia levels and documented coronary disease or strong correlation with realtive risk for coronary artery disease (CAD) and VLDL triglycerides, even contributions of elevated triglycerides as an independent risk factor for cardiovascular disease were documented in previous epidemiological studies [50, 57, 58].

In context of hypothyroidism and atherogenic dyslipidaemia induction are shown contradictory results in the literature. One study, focused on patients with overt hypothyroidism [18], found no significant difference between the prevalence of phenotype B (predominance of sdLDL) and a control group. According to other authors, the size of LDL particles is not affected by the degree of thyroid dysfunction and increased risk of atherosclerosis in hypothyroidism is probably not related to the particle size of LDL [20]. Another study was aimed to assess the relationship between atherogenic dyslipidemia, sdLDL and hypothyroidism. The results shown that underactive thyroid gland affects the size of LDLs [19].

In our study we did not notice a positive correlation between thyroid function parameters and the levels of LDL3-7 although the prevalence of LDL3-7 (phenotype B) was higher or only in hypothyroid subjects (52%), than in patients with hyperthyroidism or in normal/healthy population, which is reported to be about 31-44% [47, 59–61]. Although Abbas et al. study [19] alerts that the short-term treatment of hypothyroidism is sufficient to normalize quantitative lipid profiles, but not the qualitative lipid profiles, it is possible that the latter may need longer-term treatment with thyroxine to improve. Our results have showed that substitution treatment with levothyroxine (average dose 81.25 μg/day) during 7.13 months lead to an improvement of the lipid levels as well as LDL subclasses and LDL 3–7 (Table 4). In comparison to their treatment which lasted for a mean 15.7 weeks (range 6–40 weeks), in our study was the average treatment 28.52 weeks (range 12–36). On the other side, in our study was found a 30% prevalence of hyper-beta-lipoproteinemia in euthyroid subjects after thyreo-substitution. Probably this interval was also not adequate to normalising lipid profiles, or it is typical lipid profile for some subjects for their euthyroid state. The effect of thyroid hormones on the composition of lipoprotein spectrum and sdLDL occurence supports the fact, that in the group of patients with hyperthyroidism, LDL3-7 particles were not detected in any patient. Thyreosuppresive treatment had the opposite effect on lipoprotein levels but also without phenotype B presentation in euthyreossis (Table 5).

Overall, we cannot say that either hyperthyroidism or hypothyroidism is more favorable condition. There is an incidence of adverse effects resulting from either increased or underactive thyroid gland which can lead to higher cardiovascular morbidity and mortality and also overall morbidity. This has been shown in a several cross-sectional and prospectively studies although they were not completely uniform in its conclusions [62–64]. It only can be said, that an alteration of thyroid function leads to change in the lipid levels and lipoprotein subclasses and thus to occurrence of sdLDL in hypothyreosis in our examined group of patients, which was predominantly consisted of postmenopausal women with elevated levels of BMI. But in this context a substitution treatment of subclinical hypothyroidism by levothyroxine may also be helpful, taking into consideration the occurrence of pro-atherogenic changes in the distribution of lipid [42].

## Conclusion

The atherogenic lipoprotein profile was proved in 52.5% of patients with hypothyroidism what presents a higher prevalence than in normal or in age-related population.

Substitution treatment with levothyroxine leads to an improvement of the lipid levels, TG and as well as LDL subclasses. It significantly changed the presentation of sdLDL in lipoprotein profiles – we noticed shift from the atherogenic sdLDL to large, less atherogenic LDL particles.

SdLDL particles were not detected in any patient with hyperthyroidism and thyreosuppresive treatment led to significant rise in levels of lipid and lipoprotein subclasses. Non-atherogenic lipoprotein profile was present in group of hyperthyroid patients before and also after treatment.

Analysis of plasma atherogenesis markers: the levels of small dense LDL particles correlated less ambiguous with AIP than apoB levels; correlation between the amounts of TC or LDLs and the presence of sdLDL was not recorded; significantly positive correlation between the sdLDL and TAG and sdLDL and VLDL alerts to hypertriglyceridemia as a major cardiovascular risk factor.

We would like to point to the identification of the hyper-beta-lipoproteinemia, with LDL1, 2 non-atherogenic lipoprotein profile A among study participants.

## References

[1] Rizzo M, Berneis K (2006). Low-density lipoprotein size and cardiovascular risk assessment. QJM.

[2] Fisher WR (1983). Heterogenity of plasma low density lipoproteins: manifestation of the physiologic phenomenon in man. Metabolism.

[3] Lamarche B, Lemieux I, Despres JP (1999). The small, dense LDL phenotype and the risk of coronary heart disease: epidemiology, patho-physiology and therapeutic aspects. Diabetes Metab.

[4] Lamarche B, Tchernof A, Moorjani S, Cantin B, Dagenais GR, Lupien PJ, Després JP (1997). Small dense low-density lipoprotein particles as a predictor of the risk of ischemic heart Disease in men. Prospective results from the Quebec Cardiovascular study. Circulation.

[5] Sacks FM, Campos H (2003). Clinical review 163: Cardiovascular endocrinology 4: Low-density lipoprotein size and cardiovascular disease: a reappraisal. J Clin Endocrinol Metab.

[6] Annonymous (2001). Executive summary of the third report of The National Cholesterol Education Program (NCEP) expert panel on detection, evaluation, and treatment of high blood cholesterol in adults (Adult Treatment Panel III). JAMA.

[7] Berneis KK, Kraus RM (2002). Metabolic origins and clinical significance of LDL heterogenity. J Lipid Res.

[8] Rizzo M, Berneis K, Corrado E, Novo S (2006). The significance of low-density-lipoproteins size in vascular disease. Int Angiol.

[9] Nielsen LB (1996). Transfer of low density lipoprotein into the arterial wall and risk of atherosclerosis. Atherosclerosis.

[10] Anber V, Millar JS, McConnell M, Shepherd J, Packard CJ (1997). Interaction of very-low-density, intermediate-density, and low-density lipoproteins with human arterial wall proteoglycans. Arterioscler Thromb Vasc Biol.

[11] Austin MA, King MC, Vranizan KM, Krauss RM (1990). Atherogenic lipoprotein phenotype. A proposed genetic marker for coronary heart disease risk. Circulation.

[12] Krauss RM (2001). Atherogenic lipoprotein phenotype and diet-gene interactions. J Nutr.

[13] Rizzo M, Berneis K (2006). Should we measure routinely the LDL peak particle size. Int J Cardiol.

[14] Maki KC, Davidson MH, Marx P, Cyrowski MS, Maki A (2000). Association between elevated plasma fibrinogen and the small, dense low-density lipoprotein phenotype among postmenopausal women. Am J Cardiol.

[15] Festa A, D'Agostino R, Mykkanen L, Tracy R, Howard BV, Haffner SM (1999). Low-density lipoprotein particle size is inversely related to plasminogen activator inhibitor-1 levels. The Insulin Resistance Atherosclerosis Study. Arterioscler Thromb Vasc Biol.

[16] Jellinger PS, Dickey RA, Ganda OP, Mehta AE, Nguyen TT, Rodbard HW, Seibel JA, Shepherd MD, Smith DA (2000). The american association of clinical endocrinologists, medical guidelines for clinical practice for the diagnosis and treatment of dyslipidemia and prevention of atherogenesis. Endocr Pract.

[17] Packard CJ (2003). Triacylglycerol-rich lipoproteins and the generation of small, dense low-density lipoprotein. Biochem Soc Trans.

[18] Roscini AR, Lupattelli G, Siepi D, Pagliaricci S, Pirro M, Mannarino E (1999). Low-density lipoprotein size in primary hypothyroidism. Effects of hormone replacement therapy. Ann Nutr Metab.

[19] Abbas JMK, Chakraborty J, Akanji AO, Doi SAR (2008). Hypothyroidism results in small dense LDL independent of IRS Traits and hypertriglyceridemia. Endocr J.

[20] Kim CS, Kang JG, Lee SJ, Ihm SH, Yoo HJ, Nam JS, Ahn CW, Kim KR (2009). Relationship of low-density lipoprotein (LDL) particle size to thyroid function status in Koreans. Clin Endocrinol (Oxf).

[21] Duntas LH, Brenta G (2012). The effect of thyroid disorders on lipid levels and metabolism. Med Clin North Am.

[22] Ittermann T, Baumeister SE, Völzke H, Wasner C, Schminke U, Wallaschofski H, Nauck M, Lüdemann J (2012). Are serum TSH levels associated with oxidized low-density lipoprotein? Results from the Study of Health in Pomerania. Clin Endocrinol (Oxf).

[23] Regmi A, Shah B, Rai BR, Pandeya A (2010). Serum lipid profile in patients with thyroid disorders in central Nepal. Nepal Med Coll J.

[24] Teixeira Pde F, Reuters VS, Ferreira MM, Almeida CP, Reis FA, Buescu A, Costa AJ, Vaisman M (2008). Lipid profile in different degrees of hypothyroidism and effects of levothyroxine replacement in mild thyroid failure. Transl Res.

[25] Dobiasova M, Frohlich J (2001). The plasma parameter Log (TG/HDL-C) as an atherogenic index: correlation with lipoprotein particle size and esterification rate in apo-B-lipoprotein-depleted plasma (FER_HDL_). Clin Biochem.

[26] Dobiasova M (2004). Atherogenic index of plasma [Log (Triglycerides/HDL-Cholestrol)]: Theoretical and practical implications. Clin Chem.

[27] Rainwater DL, Moore PH, Shelledy WR, Dyer TD, Slifer SH (1997). Characterization of a composite gradient gel for the electrophoretic separation of lipoproteins. J Lipid Res.

[28] Hirano T, Ito Y, Yoshino G (2005). Measurement of small dense low-density lipoprotein particles. J Atheroscler Thromb.

[29] Otvos JD, Jeyarajah EJ, Bennett DW, Krauss RM (1992). Development of a proton nuclear magnetic resonance spectroscopic method for determining plasma lipoprotein concentrations and subspecies distributions a single, rapid measurement. Clin Chem.

[30] Friedewald WT, Levy RI, Frederickson DS (1972). Estimation of the concentration of low density lipoprotein cholesterol in plasma, without use of the preparative ultracentrifuge. Clin Chem.

[31] Hoefner DM, Hodel SD, O’Brien JF, Branum EL, Sun D, Meissner I, McConnell JP (2001). Development of a rapid quantitative method for LDL subfraction with use of the Quantimetrix Lipoprint LDL system. Clin Chem.

[32] Hirany SV, Othman Y, Kutscher P, Rainwater DL, Jialal I, Devaraj S (2003). Comparison of low-density lipoprotein size by polyacrylamide tube gel electrophoresis and polyacrylamide gradient gel electrophoresis. Am J Clin Pathol.

[33] Ensign W, Hill N, Heward CB (2006). Disparate LDL phenotypic classification among 4 different methods assessing LDL particle characteristics. Clin Chem.

[34] Van J, Pan J, Charles MA, Krauss R, Wong N, Wu X (2007). Atherogenic lipid phenotype in a general group of subjects. Arch Pathol Lab Med.

[35] Gazi I, Lourida ES, Filippatos T, Tsimihodimos V, Elisaf M, Tselepis AD (2005). Lipoprotein-associated phospholipase A2 activity is a marker of small, dense LDL particles in human plasma. Clin Chem.

[36] Austin MA, Hokanson JE, Brunzell JD (1994). Characterization of low-density lipoprotein subclasses: methodologic approaches and clinical relevance. Curr Opin Lipidol.

[37] Fruchart JCH, Sacks FM, Hermans MP, Assman G, Brown WV, Ceska R, Chapman MJ, Dodson PM, Fioretto P, Ginsberg HN, Kadowaki T, Lablanche JM, Marx N, Plutzky J, Reiner Z, Rosenson RS, Staels B, Stock JK, Sy R, Wanner C, Zambon A, Zimmet P (2008). The Residual Risk Reduction Initiative: a call to action to reduce residual vascular risk in dyslipidaemic patients. Diab Vasc Dis Res.

[38] Jenkins AJ, Rowley KG, Lyons TJ, Best JD, Hill MA, Klein RL (2004). Lippoproteins and diabetic microvascular complications. Curr Pharm Des.

[39] Assmann G (2006). Dyslipidemia and global cardiovascular risk: clinical issues. Eur Heart J Suppl.

[40] Kato M, Dote K, Sasaki S, Ueda K, Kono Y, Naganuma T, Watanabe Y, Kajikawa M, Yokoyama H, Higashi A (2009). Clinical impact of dyslipidemia for coronary plaque vulnerability in acute coronary syndrome without metabolic syndrome. J Cardiol.

[41] St-Pierre AC, Cantin B, Dagenais GR, Mauriege P, Bernard PM, Després JP, Lamarche B (2005). Low-density-lipoprotein subfraction and the long-therm risk of ischemic heart disease in men: 13-year follow-up data from the Quebec Cardiovascular Study. Arterioscler Thromb Vasc Biol.

[42] Jiskra J, Limanovi Z, Antosova M (2007). Thyroid diseases, dyslipidemia and cardiovascular risk. Vnitr Lek.

[43] Hak AE, Pols HA, Visser TJ, Drexhage HA, Hofman A, Witteman JC (2000). Subclinical hypothyreoidism is an independent risk factor for atherosclerosis and myocardial infarction in erderly women: the Rotterdam Study. Ann Intern Med.

[44] Imaizumi M, Akahoshi M, Ichimaru S, Nakashima E, Hida A, Soda M, Usa T, Ashizawa K, Yokoyama N, Maeda R, Nagataki S, Eguchi K (2004). Risk for ischemic heart disease and all-cause mortality in subclinical hypothyroidism. J Clin Endocrinol Metab.

[45] Walsh JP, Bremner AP, Bulsara MK, O’Leary P, Leedman PJ, Feddema P, Michelangeli V (2005). Subclinical thyroid dysfunction as a risk factor for cardiovascular disease. Arch Intern Med.

[46] Wanjia X, Chenggang W, Aihong W, Xiaomei Y, Jiajun Z, Chunxiao Y, Jin X, Yinglong H, Ling G (2012). A high normal TSH level is associated with an atherogenic lipid profile in euthyroid non-smokers with newly diagnosed asymptomatic coronary heart disease. Lipids Health Dis.

[47] Austin MA, Breslow JL, Hennekens CH, Buring JE, Willet WC, Krauss RM (1988). Low density lipoprotein subclass patterns and risk of myocardial infarction. JAMA.

[48] Oravec S, Gruber K, Dostal E, Mikl J (2011). Hyper-betalipoproteinemia LDL 1,2: A newly identified nonatherogenic hypercholesterolemia in a group of hypercholesterolemic subjects. Neuro Endocrinol Lett.

[49] Castelli WP (1988). Cholesterol and lipids in the risk of coronary artery disease – The Framingham Heart Study. Can J Cardiol.

[50] Castelli WP (1992). Epidemiology of triglycerides; a view from Framingham. Am J Cardiol.

[51] Castelli WP (1998). The new pathophysiology of coronary artery disease. Am J Cardiol.

[52] Tan MH, Johns D, Glazer NB (2004). Pioglitazone reduces atherogenic index of plasma in patients with type 2 diabetes. Clin Chem.

[53] Brewer HB (1999). Hypertriglyceridemia: changes in the plasma lipoproteins associated with an increased risk of cardiovascular disease. Am J Cardiol.

[54] Ooi EMM, Barrett PHR, Chan DC, Watts GF (2008). Apolipoprotein C-III: understanding an emerging cardiovascular risk factor. Clin Sci.

[55] Zheng C, Khoo C, Furtado J, Sacks FM (2010). Apolipoprotein C-III and the metabolic basis for hypertriglyceridemia and the dense LDL phenotype. Circulation.

[56] Sniderman AD, Rosenbloom M (2005). If apoB is so good, why isn’t everybody measuring it? One reason why we need The Netherlands Journal of Medicine!. Neth J Med.

[57] Albrink MJ, Man EB (1959). Serum triglycerides in coronary artery disease. Arch Intern Med.

[58] Sprecher DL (1998). Triglycerides as a risk factor for coronary artery disease. Am J Cardiol.

[59] Holewijn S, Sniderman AD, den Heijer M, Swinkels DW, Stalenhoef AF, de Graaf J (2011). Application and validation of a diagnostic algorithm for the atherogenic apoB dyslipoproteinemias: ApoB dyslipoproteinemias in a Dutch population-based study. Eur J Clin Invest.

[60] Austin MA, King MC, Vranizan KM, Newman B, Krauss RM (1988). Inheritance of low-density lipoprotein subclass patterns: results of complex segregation analysis. Am J Hum Genet.

[61] Campos H, Blijlevens E, McNamara JR, Ordovas JM, Posner BM, Wilson PW, Castelli WP, Schaefer EJ (1992). LDL particle size distribution. Results from the Framingham Offspring Study. Arterioscler Thromb.

[62] Biondi B (2012). How could we improve the increased cardiovascular mortality in patients with overt and subclinical hyperthyroidism?. Eur J Endocrinol.

[63] Yang LB, Jiang DQ, Qi WB, Zhang T, Feng YL, Gao L, Zhao J (2012). Subclinical hyperthyroidism and the risk of cardiovascular events and all-cause mortality: an updated meta-analysis of cohort studies. Eur J Endocrinol.

[64] Brandt F, Green A, Hegedüs L, Brix TH (2011). A critical review and meta-analysis of the association between overt hyperthyroidism and mortality. Eur J Endocrinol.

